# What Cross-morphemic Letter Transposition in Derived Nonwords Tells us about Lexical Processing

**DOI:** 10.5334/joc.39

**Published:** 2018-07-11

**Authors:** Marcus Taft, Sonny Li, Elisabeth Beyersmann

**Affiliations:** 1 UNSW, Sydney, AU; 2 Department of Cognitive Science and ARC Centre of Excellence in Cognition and its Disorders, Macquarie University, Sydney, AU

**Keywords:** Reading, Visual word processing, Word processing

## Abstract

According to an obligatory decomposition account of polymorphemic word recognition, a nonword that is composed of a real word plus derivational affix (e.g., *teachen*) should prime its stem (*TEACH*) to the same extent that a truly suffixed word does (e.g., *teacher*). The stem will be activated in both cases after the suffix is removed prior to the lexical status of the letter-string being of relevance. Importantly, disruption to the stem and suffix through letter transposition should have the same impact on the nonwords and words, with *teacehn* and *teacehr* equally priming *TEACH*. However, an experiment by Diependaele, Morris, Serota, Bertrand, and Grainger (2013) found that the equivalent priming for nonwords and words only occurred when they were intact. When letters were transposed, only the truly derived words showed priming. Since such a result cannot be handled by an obligatory decomposition account, it is important to replicate it. Therefore, the present study repeated the conditions of Diependaele et al. (2013), along with a nonword condition where the stem was followed by a non-suffix (e.g., *teachin* or *teacihn*). It was found that priming was maintained across all conditions regardless of letter transposition, hence maintaining obligatory decomposition as a viable account. However, the findings with the non-suffixed nonwords led to the conclusion that morphological structure does not control decomposition, but rather, has its impact after form-based components of the letter-string have been activated.

How do we read derivationally complex words such as *teacher*? Evidence strongly suggests that derived words are decomposed into their stem and affix early in their processing. As summarized by Rastle and Davis (2008), the presentation of a monomorphemic word as a masked prime facilitates the recognition of a word embedded at its beginning, but only if the letters following that embedded word have the appearance of an affix (e.g., *corner* primes *CORN*, but *cashew* does not prime *CASH*). This implies that words are automatically decomposed on the basis of their orthographic appearance as a derived word, even though there might be no semantic relationship between the whole word and its components as in the case of *corner* (i.e., decomposition is ‘blind’ to the lexical information associated with the word and, as such, is ‘pre-lexical’).

On the other hand, it must logically be the case that polymorphemic words possess a whole-word representation in lexical memory when their meaning cannot be entirely generated from the meaning of their individual morphemes, and this is the situation for most derivationally complex words. For example, while *teacher* has a meaning that is transparently derived from the meaning of *teach* and the agentive function of -*er*, there needs to be a whole-word representation that provides access to information about teachers that is not specifically associated with either the act of teaching or agentive function *per se* (such as the typical age, gender, educational background, or work attire of teachers). So, there exists a potential incompatibility between the existence of blind decomposition on the one hand and the requirement for whole-word representation on the other. Theoretical accounts differ in terms of the way in which they attempt to resolve this.

According to the strictest existing ‘obligatory decomposition’ account, Taft and colleagues propose that pre-lexical decomposition is the only avenue available for the recognition of derivationally complex words despite the existence of a whole-word representation (e.g., Taft, 2006, 2015; Taft & Ardasinski, 2006; Taft & Nguyen-Hoan, 2010; Taft & Nillsen, 2013; Xu & Taft, 2015). A word is recognized on the basis of a lemma unit (see Figure 1), which is an abstract representation that mediates between the form and function levels (i.e., between orthography/phonology on the one hand and meaning/syntax on the other). Importantly, a derived word possesses a lemma unit which is activated via lemmas corresponding to its component morphemes (e.g., the *teacher* lemma is activated via the *teach* and *-er* lemmas).

**Figure 1:**
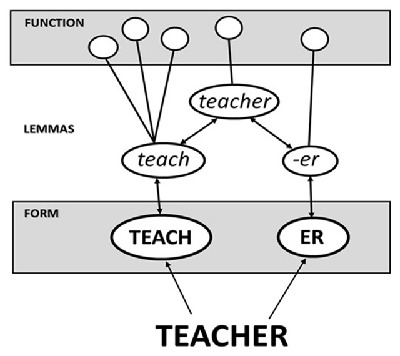
The obligatory morphological decomposition model illustrated with the derived word *teacher* being represented by a lemma that is activated via lemma units representing its component morphemes (adapted from Taft & Nguyen-Hoan, 2010).

When the word *TEACHER* is presented, letter groupings are found to overlap with the form units representing *teach* and *er* which, in turn, send activation to their corresponding lemmas that are associated with functional information relevant to them (i.e., the meaning of *teach* and the syntactic function of *-er*). It is the combined activation of these lemmas that leads, hierarchically, to activation of the lemma for *teacher*, which is associated with any further functional information relevant only to that word.

Through such pre-lexical decomposition, recognition of a word will be facilitated by the prior masked presentation of that word ending in letters that appear to form a suffix. So, not only will the presentation of *teacher* facilitate recognition of *TEACH*, but presentation of *corner* will facilitate recognition of *CORN* (e.g., Amenta & Crepaldi, 2012; Rastle & Davis, 2008). Moreover, presentation of an inappropriately suffixed nonword such as *teachen* will facilitate recognition of its stem (*TEACH*) relative to a control condition where the stem and affix have been disrupted through letter substitution (e.g., *teacsin*, an ‘SL’ nonword). Such nonword priming has been amply demonstrated (e.g., Beyersmann, Cavalli, Casalis, & Colé, 2016; Longtin & Meunier, 2005; McCormick, Rastle, & Davis, 2009).

The main alternative to the notion that the recognition of derived words must always take place through decomposition are dual-pathway models that allow decomposition and direct whole-word access to occur in parallel (e.g., Baayen, Dijkstra, & Schreuder, 1997; Bertram, Schreuder, & Baayen, 2000; Colé, Beauvillain, & Segui, 1989; Diependaele, Morris, Serota, Bertrand, & Grainger, 2013; Niswander, Pollatsek, & Rayner, 2000; Grainger & Beyersmann, 2017; Grainger & Ziegler, 2011). These models propose that a pathway incorporating pre-lexical (‘morpho-orthographic’) decomposition is supplemented with a pathway that accesses whole-words directly. According to the version proposed by Diependaele et al. (2013) and depicted in Figure 2, the latter pathway also allows for decomposition, but with a semantically based post-lexical locus by means of which only genuinely derived words are decomposed (the ‘morpho-semantic’ level of Figure 2). Moreover, according to this account (see also Grainger & Ziegler, 2011), the two pathways differ in terms of the precision with which letter information is used to access the relevant representation in lexical memory. The morpho-orthographic decompositional pathway requires fine-grained processing whereby the letters that make up the stimulus are entered into the system strictly in terms of the position in which they occur. In contrast, the whole-word pathway (depicted in the figure with a stronger line) is coarse-grained such that the exact position of a letter within the stimulus is less important than its identity, which means that a word can be activated even when its letters are out of position.

**Figure 2:**
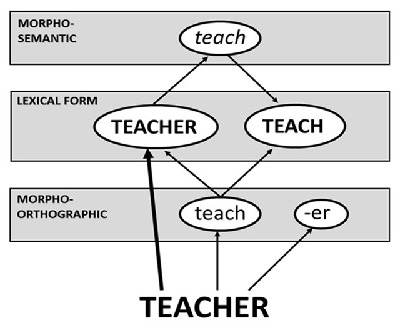
The dual-pathways model of Diependaele et al. (2013) where a whole-word lexical form for *teacher* is activated in parallel with morpho-orthographic decomposition. Post-lexical decomposition also occurs at the morpho-semantic level. The lexical level only represents existing words. Morpho-orthographic decomposition requires precise processing of letter position while whole-word access does not.

It follows from the dual-pathways model of Diependaele et al. (2013) that disruption to a suffix though letter transposition will incapacitate the fine-grained pathway. For example, when the transposed-letter (or ‘TL’) nonword *teacehr* is presented, the lexical representation for *teacher* will be activated to a large extent, but only through the whole-word pathway where precise letter position is unimportant. The disruption of the suffix means that pre-lexical decomposition cannot occur, hence precluding activation of *teacher* via morpho-orthographic decomposition. However, morpho-semantic decomposition can take place after the whole-word representation has been activated via coarse-grained processing, which means that presentation of *teacehr* as a masked prime should still facilitate recognition of the target word *TEACH*, just as the intact version of *teacher* does, and this is the observed result (e.g., Beyersmann, McCormick, & Rastle, 2013; Diependaele et al., 2013; Rueckl & Rimzhim, 2011).

In contrast, while the masked presentation of an inappropriately suffixed nonword (e.g., *teachen*) facilitates recognition of its stem, disruption to the suffix through transposition should prevent such priming. That is, recognition of *TEACH* should be no faster when preceded by *teacehn* than by the SL control *teacisn*. Diependaele et al. (2013) predict such a result because the disrupted suffix prevents the fine-grained pathway from accessing *teach*, while the fact that *teachen* is a nonword means that the coarse-grained pathway is unable to activate a whole-word representation, hence preventing post-lexical decomposition. Thus, there is no avenue through which the representation of the stem *teach* can be pre-activated when *teacehn* is presented as a masked prime.

Diependaele et al. (2013) reported results that they took as support for this prediction. When the primes were intact, both derived words (e.g., *teacher*) and derived nonwords (e.g., *teachen*) equally facilitated lexical decision responses to their stem (i.e., *TEACH*) relative to an unrelated word prime (e.g., *coastal*). However, when letters were transposed across the morpheme boundary, there was no longer any facilitation for the nonword items. That is, *teacehr* still generated priming of *TEACH* relative to the unrelated prime *coasatl*, but *teacehn* did not.

Such a result, if upheld, constitutes a major problem for the obligatory decomposition model. If only pre-lexical decomposition were available, the fact that the TL prime *teacehr* facilitates recognition of *TEACH* must mean that decomposition is able to take place after inexact letter assignment. That is, decomposition can only occur if the *h* is treated as though it occurs in the fifth letter slot rather than in the sixth, and vice versa for the second *e*, because such inexact letter assignment will then correspond to a version of the letter string that has a decomposable stem-plus-suffix structure (i.e., *teacher*). So, the existence of TL priming shows that pre-lexical decomposition can still take place even when the stem and affix are disrupted through the transposition of letters (see Taft & Nillsen, 2013 for a further discussion of how TL effects might be incorporated into the obligatory decomposition model). Importantly, exactly the same situation should hold for derived nonwords. That is, the same imprecise assignment of letters to slots should lead to the treatment of *teacehn* as *teachen* and, therefore, the decomposition that occurs for the latter should also occur for the former. As such, there is no mechanism available to the obligatory decomposition model to explain the lack of TL priming for derived nonwords as reported by Diependaele et al. (2013).

Before abandoning the obligatory decomposition model, however, it is necessary to confirm the disappearance of priming for derived nonwords when letters are transposed across the morpheme boundaries. After all, there was a 12 ms trend toward priming for the TL nonwords in the Diependaele et al. (2013) study, which is not so different in magnitude to the 19 ms priming that was significant for the intact nonwords. In fact, the intact and TL primes were analyzed separately and there is no mention of whether letter disruption significantly interacted with priming. From the reported data, it seems unlikely that it would have.

On the other hand, the TL words in the Diependaele et al. (2013) study generated significantly greater priming than their TL nonwords (with a 25 ms difference between *teacehr-TEACH* and *teacehn-TEACH*), while there was no significant difference between the words and nonwords when the prime was intact (with only a 6 ms advantage for *teacher-TEACH* over *teachen-TEACH*). Again, the interaction was not statistically tested, but a 19 ms difference in the relative magnitude of priming is nevertheless substantial. If such an interaction were indeed genuine, the obligatory decomposition account would be hard to sustain because, according to that account, both letter processing and decomposition take place before the lexical status of the derivationally complex letter-string is known and, therefore, whatever impact letter transposition has on the magnitude of priming should be the same for words and nonwords. The results of Diependaele et al. (2013) suggest that it might not be.

Having said that, however, there is something unusual about the results that Diependaele et al. (2013) report for the TL word condition (e.g., *teacehr-TEACH*), with the mean RT being considerably shorter than logic would dictate. In particular, responses in this condition were 15 ms faster than in the intact word condition (*teacher-TEACH*), despite there being no rational grounds for a disrupted prime to generate greater priming than an intact prime. If anything, one would expect more priming from an intact word than a disrupted one because the former has a more precise overlap of letters between the prime and target. Indeed, consistent with this expectation, Diependaele et al. (2013) reported another experiment in which they included both an intact and TL derived-word condition and, this time, found a significant 10 ms advantage for the former over the latter. The fact that their two experiments showed a completely contradictory pattern of results when comparing intact and TL derived words was not addressed by Diependaele et al. (2013), but raises concerns about the reliability of their data. Such concerns are reinforced by the fact that each participant contributed a maximum of only 10 responses per condition in the experiment that showed greater priming for the TL than the intact word primes, and responses from a maximum of only 12 participants were collected for each item in each condition.

It can be seen, then, that it is still an open question whether priming for derived nonwords does indeed disappear under TL conditions, hence rendering the obligatory decomposition model untenable. While the results of Diependaele et al. (2013) suggest that this is the case, their data can be called into question. For this reason, the present study again compares derived words and nonwords under both intact and TL conditions, but with a larger set of items and participants. The critical question is whether presentation of a masked derived nonword facilitates the subsequent recognition of its stem even when the prime is disrupted through letter transposition across the stem-affix boundary.

In addition to the two conditions where the intact version of the prime has a derivational suffix (i.e., *teacher-TEACH* and *teachen-TEACH*), a further experimental condition is included, namely, where the stem is followed by a non-suffix (e.g., *teachin-TEACH*). This condition serves to confirm previous studies that have observed priming for any nonword in which the target word is embedded at the beginning (e.g., Beyersmann, Casalis, Ziegler, & Grainger, 2014; Beyersmann et al, 2016; Beyersmann & Grainger, 2017; Hasenäcker, Beyersmann, & Schroeder, 2016; Morris, Porter, Grainger, & Holcomb, 2011), whereby *teachin* should facilitate responses to *TEACH* relative to the SL control *teacsin*. What we do not know, however, is whether such priming disappears when the embedded word is disrupted through transposition (i.e., *teacihn-TEACH*). If this does occur for both the non-suffixed and suffixed nonwords, but not the real derived words, then it will strongly suggest that only post-lexical decomposition is available when a letter-string is disrupted through transposition because it would only be shown when the whole word can be accessed. On the other hand, if priming is maintained for both of the nonword TL conditions relative to the corresponding SL control (*teacisn-TEACH*), it would mean that imprecise letter processing, however envisaged, allows the embedded word to be activated regardless of the morphological status of the letters that follow it.

Note that the existence of priming when the target is combined with a non-suffix in the prime (i.e., *teachin-TEACH*) indicates that form-based decomposition of a letter-string is not specifically determined by the morphological status of its subunits. The implications of this important point will be considered in greater detail after the pattern of priming for both intact and TL primes has been established.

The first experiment to be reported examines the pattern of masked priming when the primes are intact. While the primary focus of the study is on the impact of using primes that have a cross-morphemic letter transposition, it is necessary to first establish a baseline against which such TL effects can be compared. The intact primes of Experiment 1 provide such a baseline.

## Experiment 1 (Intact Primes)

### Method

#### Materials

There were 60 target words ranging in length from 4 to 9 letters (e.g., *TEACH, EARTH, SEGMENT, GYMNAST*) preceded by four different types of prime. In the Real Word condition, the prime was a word derived from the target by the addition of a two-letter suffix (e.g., *teacher, earthen, segmental, gymnastic*). In the Derived Nonword condition, the same set of suffixes was re-paired with a stem of the same length in such a way that a nonword was created (e.g., *teachen, earther, segmentic, gymnastal*). The third condition (Non-Derived Nonwords) was created by replacing the first letter of the suffix in the Derived Nonword condition to create a non-existing suffix (e.g., *teachin, earthur, segmentoc, gymnastol*). Finally, the SL Control condition was generated from the Derived Nonword condition by substituting the last letter of the stem and first letter of the suffix with two different letters, hence eliminating the whole target word from the beginning of the prime (*teacsin, eartsir, segmenkoc, gymnaskol*). The use of such a control condition would make it harder, if anything, to detect priming than the control condition adopted by Diependaele et al. (2013) where all letters differed between the prime and target. This should therefore make any evidence for priming all the more convincing in the current study. The complete set of items can be found in the Appendix.

A Latin Square design was adopted whereby participants were assigned to one of four subgroups that received 15 of the targets under each of the priming conditions, but where no participant received the same target more than once.

In addition to the 60 word targets preceded by their prime, there were 60 nonword targets each preceded by a prime that corresponded to one of the conditions used with the word targets. The target was always one or two letters different from the stem on which the prime was based. So, 15 of the items had a real word prime composed of a word with a two-letter derivational suffix (e.g., *reviewer-REVIEN*), 15 had a nonword prime also composed of a word with a two-letter suffix (e.g., *preachen-PREATH*), 15 had a nonword prime composed of a word with a two-letter non-suffix (e.g., *directoc-DIRENT*), and 15 had a nonword prime composed of a nonword created from a word by changing the final letter, followed by a two-letter non-suffix (e.g., *shrungir-SPRUNK*) where, like the SL Control condition, the target was one more letter different to the beginning of the prime than was the target in the other three conditions.

#### Procedure

Participants were tested individually in a quiet room with stimuli presented in the centre of a LCD computer screen using DMDX software (Forster & Forster, 2003). Each trial consisted of a 500-ms forward mask of hash-marks, then the prime presented in lowercase for 50 ms, followed by the target presented in uppercase. The target was flanked by two equal signs on each side in order to ensure that it fully masked the prime despite being shorter in length (something that was not done in the Diependaele et al., 2013, study). The target remained on-screen until a response was given, up to a maximum of 4000 ms. Trials were presented in a random order with an inter-trial interval of 1000 ms, with participants being instructed to decide as quickly but as accurately as possible by key-press whether the uppercase letter-string was a real English word or not. There were 12 practice items that corresponded in structure to the items used in the actual experiment.

#### Participants

The participants were 69 students from UNSW Sydney, all native English speakers, who received either monetary compensation ($15/hour) or course credit. Ethics approval for the research was provided by the UNSW Human Research Ethics Advisory Panel C: Psychology (File 2713).

### Results

**Figure 3:**
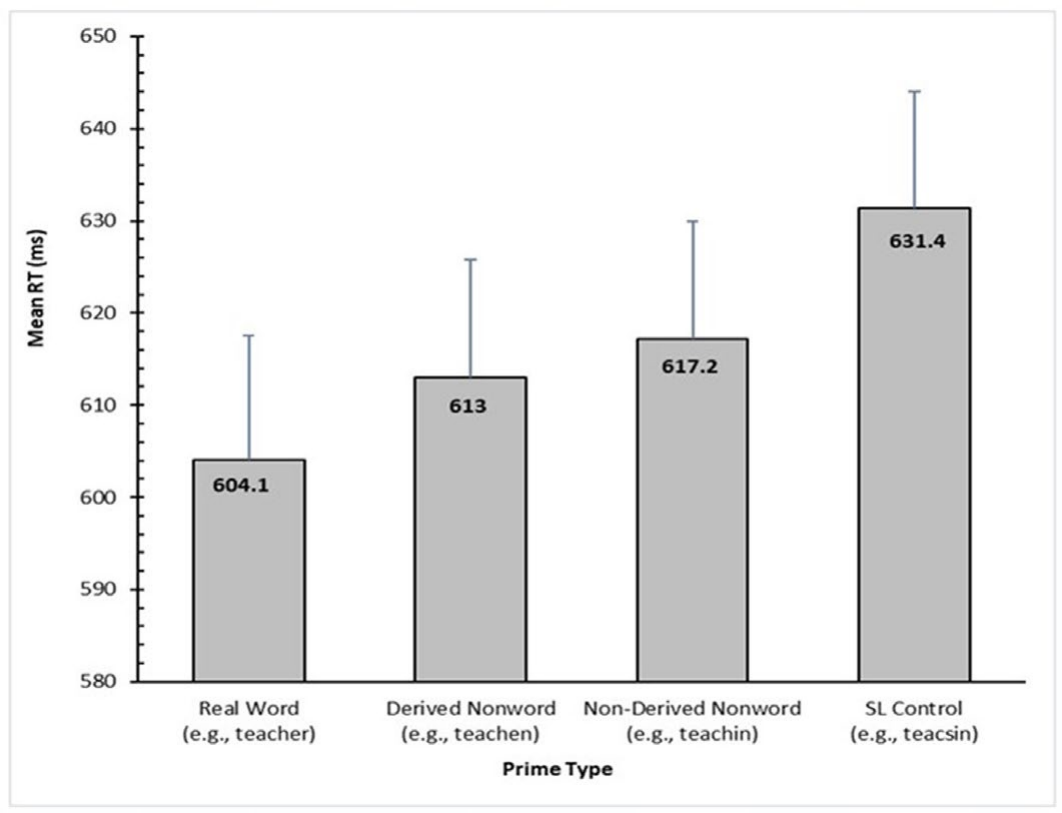
Adjusted condition means for RT (in ms) based on the final LME model for Experiment 1. Error bars represent standard error.

Response times that were faster than 200 ms and longer than 2000 ms were omitted. The item *INTERWOVE* was removed from the analysis since it was inadvertently included as a word despite being a nonword and, unsurprisingly, led to fewer than 33% classifications as a word. The RT data of one participant was also removed owing to an error rate of more than 25%. Mean RTs for correct responses from the four conditions are presented in Figure 3.

Response times were analyzed using linear mixed effects modelling in R, with the package *lme4* (Baayen, Davidson, & Bates, 2008). To minimize effects of positive skew in the distribution, inverse RTs were used (i.e., 1000/RT). The fixed effect was the prime condition (i.e., Real Word, Derived Nonword, Non-Derived Nonword, SL Control). Target word frequency and response times on the previous trial were entered as covariates, centered according to their respective means, and both were retained in the final model as they improved its goodness-of-fit as assessed via a step-wise selection procedure. Random intercepts for subjects and items were also included in the model. The effect of subject-related variance on the fixed factor was assessed via by-subject random slopes, but this was omitted from the final model as it did not improve its fit. Based upon the fit of the data to the model, data points with absolute standardized residuals exceeding 2.5 standard deviations were removed. Values for *p* were obtained by using the R package *lmerTest* (Kuznetsova, Brockhoff, & Christensen, 2014).

With the SL Control condition as the baseline (e.g., *teacsin-TEACH*), there was a significant priming effect for all three experimental conditions: *t* = 5.12, *p* < 0.001, for the Real Word condition (e.g., *teacher-TEACH*), *t* = 4.68, *p* < 0.001, for the Derived Nonword condition (e.g., *teachen-TEACH*), and *t* = 3.91, *p* < 0.001, for the Non-Derived Nonword condition (e.g., *teachin-TEACH*). In addition, the Real Word condition was associated with shorter RTs than the Non-Derived Nonword condition, *t* = 2.36, *p* = 0.02, while the Derived Nonword condition fell in between these two conditions, not differing from either, *t* = 0.84, *p* = 0.41, and *t* = 1.02, *p* = 0.31, respectively.

The analyses of error rates (ER) followed the same procedure as the RT analyses (apart from the exclusion of RT on the previous trial as a covariate). ER was entered as a binary variable using the *glmer* function in the *lme4* package, and values for *p* were generated using the Wald *z* statistic. The mean ER for the Real Word, Derived Nonword, Non-Derived Nonword, and SL Control conditions were 5.13%, 5.74%, 5.94%, and 5.31% respectively. There were no significant effects, all *p*’s > 0.50.

### Discussion

It is apparent that the embedding of the target word at the beginning of a letter-string is sufficient for the target word to be activated regardless of the letters that follow it. Such a result is consistent with previous research (e.g., Beyersmann et al., 2014, 2016), and supports the hypothesis that embedded words are mapped onto existing orthographic whole-word representations, independent of morphological structure (Grainger & Beyersmann, 2017). However, priming was weaker when the letters following the embedded word did not form a suffix (e.g., *teachin*) than when they formed a suffix and created a real word (e.g., *teacher*).

In fact, Beyersmann et al. (2014) observed a different pattern of priming depending on language proficiency (as determined from a spelling and vocabulary test) and the present results essentially correspond to the average of their high and low proficiency patterns. In particular, Beyersmann et al. (2014) found that high proficiency was associated with equivalent priming for real words, derived nonwords, and non-derived nonwords, while lower proficiency was associated with strong priming for real words, no priming at all for non-derived nonwords, and something in between for derived nonwords. In the present study, the Non-Derived Nonwords did show significant priming, but to a lesser extent than for the Real Words, which could be seen as the combination of significant priming for higher proficiency individuals counteracted to some degree by a lack of priming for lower proficiency individuals. Similarly, the intermediate pattern of priming observed for the Derived Nonwords could be seen as the significant priming for higher proficiency individuals combining with the weak priming for lower proficiency individuals.

Thus there was evidence in Experiment 1 for activation of an embedded word on purely orthographic grounds, but also for a certain amount of morphological sensitivity. Whether the same pattern of results is maintained when the primes are disrupted through letter transposition is explored in Experiment 2. According to the results of Diependaele et al. (2013) in support of a dual-pathways account, the only TL condition to show a priming effect should be the Real Words.

## Experiment 2 (TL Primes)

### Method

#### Materials & Procedure

The items from Experiment 1 were used in Experiment 2 except that every prime included a transposition of the final letter of its stem with the first letter following it. Examples of each of the four conditions are *teacehr-TEACH* (TL Real Word), *teacehn-TEACH* (TL Derived Nonword), *teacihn-TEACH* (TL Non-Derived Nonword), and *teacisn-TEACH* (SL Control). The primes used with the nonword targets also underwent transposition in the same way (e.g., *revieewr-REVIEN, preacehn-PREATH, direcotc-DIRENT, shrunigr-SPRUNK*).

The procedure was identical to that of Experiment 1.

#### Participants

The participants were 80 native English speakers who were students at UNSW Sydney. They received either monetary compensation ($15/hour) or course credit. Ethics approval for the research was provided by the UNSW Human Research Ethics Advisory Panel C: Psychology (File 2713).

### Results

**Figure 4:**
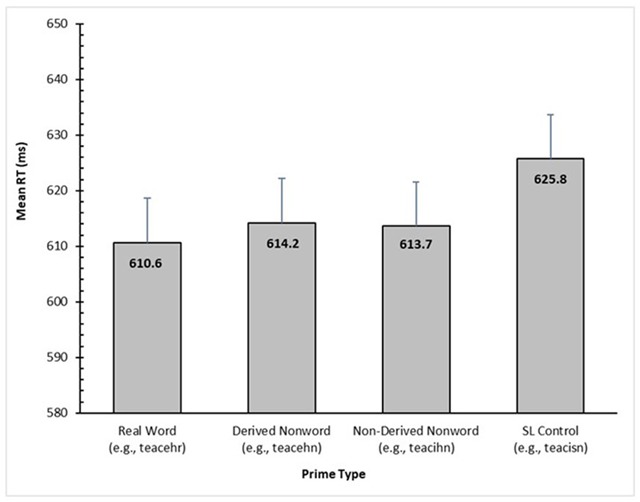
Adjusted condition means for RT (in ms) based on the final LME model for Experiment 2. Error bars represent standard error.

The data were analyzed in exactly the same way as in Experiment 1, with the same fixed factor (i.e., prime condition: Real Word, Derived Nonword, Non-Derived Nonword, SL Control), random factors, and covariates. No participants were excluded, but the item *INTERWOVE* was again removed. Mean RTs for correct responses for the four conditions are presented in Figure 4.

In terms of RTs, there was significant priming for each of the three experimental conditions relative to the SL Control condition (e.g., *teacisn-TEACH*): *t* = 3.60, *p* < 0.001, for the Real Word condition (e.g., *teacehr-TEACH*), *t* = 2.66, *p* = 0.01 for the Derived Nonword condition (e.g., *teacehn-TEACH*), and *t* = 2.66, *p* = 0.01,^1^ for the Non-Derived Nonword condition (e.g., *teacihn-TEACH*). The Real Words did not significantly differ from either the Derived Nonwords or Non-Derived Nonwords, *t* = 1.26, *p* = 0.21, and *t* = 1.33, *p* = 0.19, respectively, and neither did the latter two differ from each other, *t* = 0.16, *p* = 0.87.

The mean ER for the Real Word, Derived Nonword, Non-Derived Nonword, and SL Control conditions were 5.20%, 6.19%, 6.02%, and 6.35% respectively. There were no significant effects, all *p*’s > 0.27.

### Discussion

The results failed to replicate the lack of priming reported by Diependaele et al. (2013) for derived nonwords when their morphological structure was disrupted through transposition. Indeed, the priming observed for such items was just as strong as that observed for real derived words with a similar disruption. If it had been found that TL nonwords did not prime their stem, an obligatory decomposition model would have been hard to sustain. However, the significant priming that was observed indicates that such a model is still viable. It seems that the experiment of Diependaele et al. (2013) simply lacked the power to detect significant priming for the affixed nonwords, unlike the present study that tested more than twice their number of participants. Moreover, it also seems that the unexpectedly large magnitude of priming observed by Diependaele et al. (2013) in their TL Word condition might have been an aberration since there was no indication in the present study that the degree of priming was greater for the TL words than for the TL nonwords.

Nevertheless, the fact that priming occurred whether the disrupted nonword ended in an existing suffix or not is inconsistent with a model that centers entirely on morphologically determined form-based decomposition. How the results might be explained will be considered later.

## Cross-experiment analyses

Unlike the Diependaele et al. (2013) study, a direct examination was made here of the relative impact of the intact and TL primes. An LME analysis was carried out on RTs across the two experiments with the fixed factors being prime condition (as in the previous analyses), type of prime (Intact vs TL), and the interactions between these. The same covariates and random factors were included as in the analyses of the individual experiments. The magnitude of priming relative to the SL baseline was significantly greater with intact than disrupted primes for both the Real Word and Derived Nonword conditions, *t* = 2.22, *p* = 0.03, and *t* = 2.35, *p* = 0.02, respectively, but not for the Non-Derived Nonwords, *t* = 1.26, *p* = 0.21. No other interactions were significant, all *p*’s > 0.39.

It can be seen from this analysis that priming was stronger when both the stem and affix were intact than when they were disrupted through transposition. This simply suggests that having letters in their correct position activates lexically stored information more effectively than having them out of position. Importantly, though, the stronger facilitation for intact over TL primes was equally seen in the Derived Nonword condition as it was in the Real Word condition.

The finding that the Non-Derived Nonword primes did not have a significantly stronger impact when intact (e.g., *teachin*) than when disrupted (e.g., *teacihn*) might be ascribed to the fact that the disrupted final letters did not correspond to any lexically stored information. That is, both the disrupted stem and affix of the TL Derived Nonwords had the potential to activate existing representations, whereas this was only possible for the disrupted stem in the case of the TL Non-Derived Nonwords. It is unclear, however, whether the Non-Derived Nonword primes really did differ from the other two conditions in relation to the differential impact of intact and disrupted primes given that there were no interactions between the three experimental conditions in this regard.

## General Discussion

The cross-experiment analysis shows that the priming effect that was found in Experiment 1 for intact derived nonwords (e.g., *teachen-TEACH* vs *teacsin-TEACH*) was maintained when the component morphemes were disrupted through letter transposition (e.g., *teacehn-TEACH* vs *teacisn-TEACH*), even if of smaller magnitude. It was necessary to demonstrate the existence of such priming if an obligatory decomposition account were to be upheld. That is, the only locus for explaining TL effects in that model is in terms of imprecise letter assignment happening prior to or during pre-lexical decomposition, hence being applicable to words and nonwords alike.

Indeed, the results are problematic for the alternative dual-pathways model as envisaged by Diependaele et al. (2013). If pre-lexical decomposition can only take place when the letters of the stem and suffix are in their correct position, there is no locus for explaining the priming that was seen for disrupted nonword primes in Experiment 2. The only available pathway would be coarse-grained whole-word access and, given that nonwords have no whole-word representation, there is no avenue for the post-lexical decomposition that is available for existing derived words. As such, the stem of the derived nonword cannot be isolated and, therefore, no priming should have been observed.^2^ The fact that significant priming was observed suggests that decomposition can take place despite disruption to the affix (and stem), a result that is compatible with an obligatory decomposition account that incorporates flexibility in the processing of letter position prior to or during decomposition.

However, it is apparent that the pre-lexical decomposition that obligatorily occurs when a letter-string is presented is not determined by the morphological status of the components of that letter-string. Such a conclusion can be drawn from that fact that a word embedded at the beginning of a nonword is primed even when the final letters do not correspond to a suffix. That is, *teachin* primed lexical decision responses to *TEACH* despite the fact that *-in* is not an existing suffix, a result that replicates what has been found in previous studies testing English, French and German (e.g., Beyersmann, et al., 2014; Beyersmann et al., 2016; Beyersmann & Grainger, 2017; Hasenäcker et al., 2016; Morris et al., 2011). Grainger and Beyersmann (2017) propose that edge-aligned embedded words (e.g., *teach* in *teachin*) are mapped onto existing orthographic word representations just like any other free-standing whole word. As such, the activation of embedded words is an entirely non-morphological process: Embedded words are activated independently of whether they are accompanied by a suffix or a non-suffix, which is consistent with the present findings.

Now, interpretation of the facilitation arising from non-derived nonword primes (e.g., *teachin*) must be considered in the light of what is typically observed with real word primes (as summarized by Rastle & Davis, 2008), namely, that facilitation is absent when the letters added to the end of the target do not correspond to a suffix (e.g., *cashew-CASH*). The contrast between the impact of nonword and word primes of the same non-derivational structure implies that the lexical representation of the prime word (e.g., *cashew*) competes with, and hence inhibits, the representation of the target word (*CASH*), resulting in suppression of form-based facilitation that is seen when the prime has no lexical representation (e.g., *teachin-TEACH*). So, while *CASH* is not primed by *cashew*, it should be primed by *cashop* which has no lexical representation, implying that *cash* is activated at the form-level in both cases, but that priming is eliminated when a whole-word representation exists (i.e., for *cashew*) and generates lateral inhibition (Grainger & Beyersmann, 2017).

Note that the story is different when it comes to semantically unrelated prime-target pairs where the additional letters in the prime correspond to a suffix (e.g., *corner-CORN*). As with non-suffixed primes, the form-level representation of the embedded word will be activated (e.g., the *corn* of *corner*), but this time, the activation is maintained and results in priming. So, this is where the morphological status of the final letter-combination has its impact. What is therefore necessary is an explanation for the basis of this impact.

**Figure 5:**
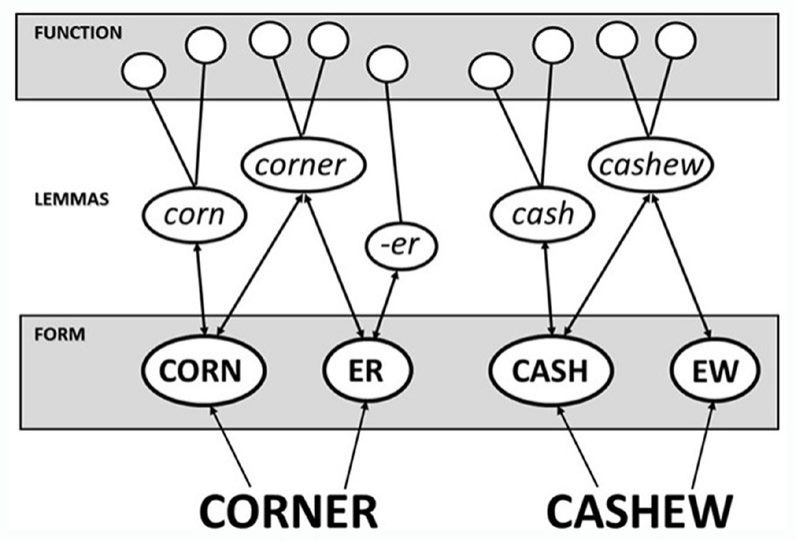
The way in which pseudo-derived words (e.g., *corner*) and non-derived words (e.g, *cashew*) might be represented within the lemma model proposed by Taft and Nguyen-Hoan (2010).

One possibility (see Taft, 2015; Taft & Nguyen-Hoan, 2010) arises from the idea that the form unit corresponding to a suffix is associated with its own lemma (as in the case of *er* in Figure 1), whereas the form unit corresponding to a non-suffix (e.g., the *ew* of *cashew*) is not associated with its own lemma. Figure 5 depicts how the system might be envisaged for the words *corner* and *cashew*. In both cases, the lemma for the embedded word (i.e., *corn* or *cash*) is activated via its representation at the form level, as is the lemma for the whole word in which it is embedded. The difference between the pseudo-derived word (i.e., *corner*) and the non-derived word (i.e., *cashew*) is whether the letters after the embedded word have a corresponding lemma or not. The potential suffix *er* does, while the non-suffix *ew* does not.

The assumption can then be made that, when all form-units within a word activate their own lemmas (as in the case of *corn* and *er*), it is worth maintaining activation in those lemmas in order to establish whether they combine to generate a genuinely affixed word (for a related account, see ‘the principle of full decomposition’ proposed by Grainger and Beyersmann, 2017). On the other hand, when a form-unit does not activate a lemma (as in the case of *ew* in *cashew*), processing is more efficient if the lemma for the embedded word (*cash*) has its activation suppressed as a result of activation in the lemma for the longer word (*cashew*).

In keeping with the idea that edge-aligned embedded words are activated at the form level (Grainger & Beyersmann, 2017), it can be seen that the obligatory decomposition model cannot treat decomposition as being strictly morphological in nature. All polysyllabic words are decomposed into smaller units that match with representations at the form level, while the impact of morphology arises at the lemma level, prior to the whole word being recognized. That is, subunits of the letter-string are activated at the form level regardless of the morphological structure of that letter-string and, in turn, each of these activates its corresponding lemma if it exists. When the letter-string is a derived word, the lemmas activated by its subunits will provide the pathway to activation of the whole-word lemma (see Figure 1). Otherwise, the whole-word lemma is directly activated by the form-level units (see Figure 5).

The idea that the morphological structure of a polysyllabic word plays its role only subsequent to orthographic decomposition is consistent with the findings of Duñabeitia, Kinoshita, Carreiras, and Norris (2011). While it is well-established that masked priming for suffixed words (e.g., *teacher-TEACH*) is greater than for non-suffixed words (e.g., *cashew-CASH*) when the task is lexical decision (e.g., Rastle & Davis, 2008), Duñabeitia et al. (2011) found equivalent masked priming for the two types of words when the task required a same-different judgement. Such a task appears to tap into pre-lexical orthographic processing, since words and nonwords show the same effects (e.g., Kinoshita & Norris, 2009). So it seems that there is pre-lexical stage of processing where morphologically complex and morphologically simple words are treated in the same way, while the morphological status of the word’s subunits only has an impact at a later stage where the masked priming effects observed with lexical decision responses arise. According to the proposed account, this later stage refers to activation at the lemma level where morphemes are represented by units and non-morphemic components are not.

Underlying the account illustrated in Figure 5 is the idea that form-units exist for components of polysyllabic words even when those components are not morphemes (e.g., *ew*), and this has been argued elsewhere to be the case (e.g., Taft, 1979, 1987; Taft & Kougious, 2004; Taft & Krebs-Lazendic, 2013). It is proposed that a word is represented by form-units that result from maximization of the coda of the first syllable (i.e., the consonants following the first vowel). For example, *sermon* is represented by the form-units *serm* and *on* (rather than *ser* and *mon*), just as *corner* is represented by *corn* and *er*, and *cashew* by *cash* and *ew*. According to Taft and Krebs-Lazendic (2013), these form-units are activated when letters are assigned to slots that correspond to the relevant onset, vowel, and coda of each component. So, in the case of *sermon*, the *s* will be assigned to the onset slot of the first component, the *e* to its vowel slot, the *r* to its first coda slot, and the *m* to its second coda slot. Since assignment to slots is imprecise, even transposed letters can lead to activation of the word. Thus, when the TL nonword *semron* is presented, the *r* and *m* will be tried out in the slots that are appropriate for activating the lemma for the word *sermon*.

Applying this idea to the findings of the present study, transposition of letters within the prime will still allow the embedded word to be activated. For example, the lemma for *teach* will be activated even when the *h* is out of position, as in *teacehr, teacehn*, and *teacihn*. This activation may not be as great as when the letter is in its correct position, but it will be sufficient to generate the priming seen in Experiment 2.

## Conclusions

The present study was designed to test the viability of an obligatory decomposition account of derived word recognition. According to such an account (exemplified here by the model of Taft and colleagues depicted in Figure 1), derived nonwords should prime lexical decision responses to their stems not only when intact (e.g., *teachen-TEACH*), but when disrupted through letter transposition (e.g., *teacehn-TEACH*), even if to a lesser degree. It was important to establish whether this was the case given that Diependaele et al. (2013) had previously presented evidence against it, with no significant priming for disrupted derived nonwords in the face of strong priming for disrupted derived real words.

In contrast to the results of Diependaele et al. (2013), it was found here that disruption of the nonword prime did not eliminate the facilitation that was observed when intact. In fact, the amount of facilitation was the same as that observed for derived real words regardless of whether the prime was intact or disrupted. Such a result is not only consistent with the obligatory decomposition approach, but argues against the dual-pathways model proposed by Diependaele et al. (2013) where decomposition requires the precise processing of letter position.

## Additional File

The additional file for this article can be found as follows:

10.5334/joc.39.s1 Appendix. List of experimental items.

## Data Availability

The data can be found at osf.io/che7r in the form of a CSV file.

## Footnotes

The stems of 14 of the 60 primes, when intact, ended in an *e* while the suffix also began with an *e* (e.g., the Derived Nonwords *smoken, refuger, managee*). In such cases, letter transposition actually resulted in what could be seen as an intra-stem transposition (e.g., *smoek* in *smoekn*). Since this may have contributed to the priming that was observed for the Derived Nonwords, a post-hoc analysis was carried out without these items, but the magnitude of the priming effect remained the same.

It is not entirely clear why Diependaele et al. (2013) dismiss the possibility that the coarse-grained pathway allows activation of a word embedded in a TL item (e.g., *teach* being activated in *teacehr* or *teacehn*). After all, every letter of the embedded word is contained within the TL stimulus, even if there are additional non-overlapping letters. Instead, it seems to be assumed that it is necessary to have all letters of the stimulus overlapping with those of a whole word if activation is going to occur (as is the case for *teacehr*, but not *teacehn*). Without such an assumption, there would have been no basis for Diependaele et al. (2013) predicting the absence of priming for the TL Derived Nonwords and interpreting their results in the way that they did.
