# Supplementary material for: What Cross-morphemic Letter Transposition in Derived Nonwords Tells us about Lexical Processing
Source: J Cogn. 2018 Jul 11;1(1):36. doi: 10.5334/joc.39 (PMC6634602; doi:10.5334/joc.39)
Supplement: Appendix. — List of experimental items. [file joc-1-1-39-s1.pdf]

Appendix

The following are the items used in Experiment 1 in the order: Real Word, Derived Nonword, Non-Derived Nonword, SL Control, and target (in uppercase). The primes used in Experiment 2 were the same except that the final letter of the embedded target word (or the equivalent letter in the SL Control) was transposed with the letter that followed it.

teacher, teachen, teachin, teacsin, TEACH; earthen, earther, earthir, eartsir, EARTH; killer, killen, killun, kilpun, KILL; fallen, faller, fallur, falpur, FALL; teaser, teasen, teasun, teadun, TEASE; chosen, choser, chosur, chodur, CHOSE; smoker, smoken, smokun, smolun, SMOKE; spoken, spoker, spokur, spolur, SPOKE; raider, raiden, raidun, raikun, RAID; wooden, wooder, woodur, wookur, WOOD; starter, starten, startin, starbin, START; hearten, hearter, heartir, hearbir, HEART; sprinter, sprinten, sprintun, sprinlun, SPRINT; frighten, frighter, frightur, frighlur, FRIGHT; braver, braven, bravun, brajun, BRAVE; frozen, frozer, frozur, frojur, FROZE; sender, senden, sendin, sentin, SEND; golden, golder, goldir, goltir, GOLD; promoter, promoten, promotun, promopun, PROMOTE; mistaken, mistaker, mistakur, mistapur, MISTAKE; wholesaler, wholesalen, wholesalun, wholesabun, WHOLESALE; interwoven, interwover, interwovur, interwobur, INTERWOVE; researcher, researchen, researchin, researcsin, RESEARCH; strengthen, strengther, strengthir, strengtsir, STRENGTH; absentee, absenter, absentir, absendir, ABSENT; exporter, exportee, exportie, expordie, EXPORT; licensee, licenser, licensur, licenlur, LICENSE; composer, composee, composue, compolue, COMPOSE; refugee, refuger, refugir, refudir, REFUGE; manager, managee, managie, manadie, MANAGE; trustee, truster, trustur, trushur, TRUST; toaster, toastee, toastue, toashue, TOAST; poetic, poetor, poetur, poelur, POET; debtor, debtic, debtuc, debluc, DEBT; segmental, segmentic, segmentoc, segmenkoc, SEGMENT;

gymnastic, gymnastal, gymnastol, gymnaskol, GYMNAST; oppressor, oppressal, oppressil, opprestil, OPPRESS; dismissal, dismissor, dismissir, dismistir, DISMISS; magnetic, magnetor, magnetar, magnesar, MAGNET; inventor, inventic, inventac, invensac, INVENT; robotic, robotor, robotur, robonur, ROBOT; visitor, visitic, visituc, visinur, VISIT; optional, optionic, optionuc, optioduc, OPTION; rhythmic, rhythmal, rhythmul, rhythdal, RHYTHM; cultural, culturic, culturoc, cultunoc, CULTURE; theatric, theatral, theatrol, theatnol, THEATRE; seasonal, seasonor, seasonur, seasobur, SEASON; governor, governal, governul, goverbul, GOVERN; anecdotal, anecdotic, anecdotuc, anecdobuc, ANECDOTE; parasitic, parasital, parasitul, parasibul, PARASITE; acidic, acidal, acidol, acipol, ACID; feudal, feudic, feudoc, feupoc, FEUD; forestry, forestly, forestvy, foreskvy, FOREST; cowardly, cowardry, cowardvy, cowarkvy, COWARD; puppetry, puppetly, puppetvy, puppedvy, PUPPET; recently, recentry, recentvy, recendvy, RECENT; dentistry, dentistly, dentistny, dentishny, DENTIST; perfectly, perfectry, perfectny, perfechny, PERFECT; mimicry, mimicly, mimicny, mimisny, MIMIC; rapidly, rapidry, rapidny, rapisny, RAPID
